# Modeling and Development of INS-Aided PLLs in a GNSS/INS Deeply-Coupled Hardware Prototype for Dynamic Applications

**DOI:** 10.3390/s150100733

**Published:** 2015-01-05

**Authors:** Tisheng Zhang, Xiaoji Niu, Yalong Ban, Hongping Zhang, Chuang Shi, Jingnan Liu

**Affiliations:** GNSS Research Center, Wuhan University, No 129 Luoyu Road, Wuhan 430079, China; E-Mails: zts@whu.edu.cn (T.Z.); xjniu@whu.edu.cn (X.N.); ylban@whu.edu.cn (Y.B.); shi@whu.edu.cn (C.S.); jnliu@whu.edu.cn (J.L.)

**Keywords:** GNSS/INS deeply-coupled integration, INS-aided PLLs, PLL tracking error, steady state error model, hardware prototype

## Abstract

A GNSS/INS deeply-coupled system can improve the satellite signals tracking performance by INS aiding tracking loops under dynamics. However, there was no literature available on the complete modeling of the INS branch in the INS-aided tracking loop, which caused the lack of a theoretical tool to guide the selections of inertial sensors, parameter optimization and quantitative analysis of INS-aided PLLs. This paper makes an effort on the INS branch in modeling and parameter optimization of phase-locked loops (PLLs) based on the scalar-based GNSS/INS deeply-coupled system. It establishes the transfer function between all known error sources and the PLL tracking error, which can be used to quantitatively evaluate the candidate inertial measurement unit (IMU) affecting the carrier phase tracking error. Based on that, a steady-state error model is proposed to design INS-aided PLLs and to analyze their tracking performance. Based on the modeling and error analysis, an integrated deeply-coupled hardware prototype is developed, with the optimization of the aiding information. Finally, the performance of the INS-aided PLLs designed based on the proposed steady-state error model is evaluated through the simulation and road tests of the hardware prototype.

## 1. Introduction

The tracking loop design of the traditional global navigation satellite system (GNSS) receiver is suffering a dilemma. In order to reduce the thermal noise to improve the signal receiving accuracy in the GNSS receiver, the bandwidth of its tracking loops should be narrow enough. However, the narrow bandwidth would increase dynamic stress error [1]. Therefore, receiver measurement errors during dynamic periods are systematically larger than those during static periods [2]. The inertial navigation system (INS) has a superior dynamic characteristic, which is highly complementary to GNSS. GNSS/INS integration can be briefly classified as loosely-coupled, tightly-coupled and deeply-coupled, listed in order of complexity. While GNSS navigation results are fused with INS information in the loosely-coupled integration, GNSS observation results are fused with INS information in the tightly-coupled integration. The GNSS receiver's baseband structure need not be adjusted in loosely-coupled integration and tightly-coupled integration, but satellite signal receiving performance cannot be improved. The deeply-coupled integration is the fusion of GNSS and INS information in the signal processing level, which could further take advantage of the INS dynamic characteristic to improve satellite signal acquisition and tracking performances under dynamics [3].

**Figure 1.**
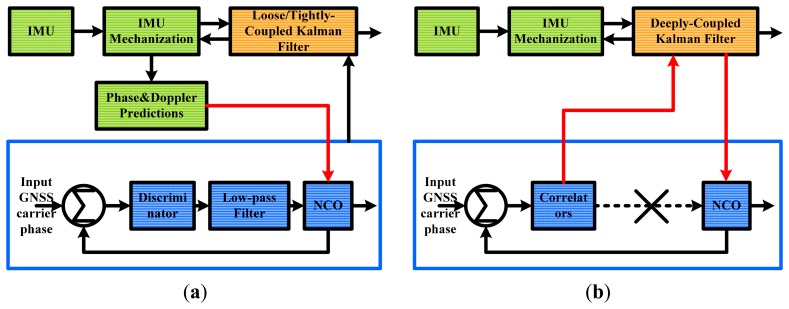
Two different architectures of GNSS/INS deeply-coupled integration. (**a**) Scalar-based architecture; (**b**) vector-based architecture. NCO means numerically controlled oscillator.

Based on the type of tracking loops used in receivers, deeply-coupled integration can be implemented in two different ways, which are shown in Figure 1, respectively named: (1) scalar-based architecture; and (2) vector-based architecture [4]. The term “scalar-based architecture” refers to aiding the individual tracking loops by inertial measurements, proposed by Stanford University [5]. By contrast, the term “vector-based architecture” is considered as a vector-based receiver integrated with an inertial measurement unit (IMU), in which the traditional code and carrier tracking loops are eliminated, proposed by MIT [6]. Although the vector-based architecture can make fuller use of the available information and get better signal sensitivity, the architecture is more complex and challenging for realizing a real-time system. Meanwhile, since the deeply-coupled Kalman filter output accuracy is insufficient for carrier phase tracking, individual tracking loops still need be used in the vector-based architecture [7]. As a simpler approach, the scalar-based architecture, whose tracking loops are individual, is valuable for carrier phase tracking.

**Figure 2.**
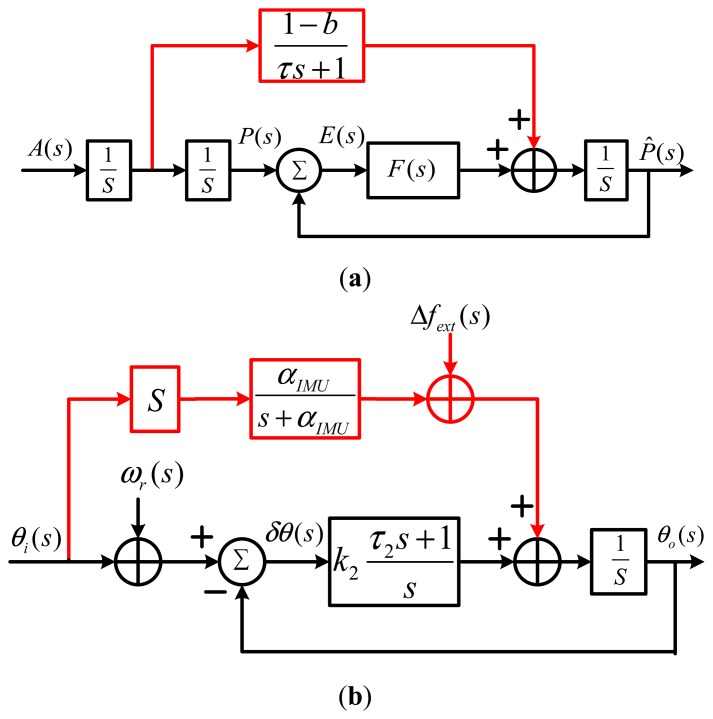
INS-aided GNSS receiver tracking loop model in previous work [9,10]. (**a**) Proposed by He *et al.*; (**b**) proposed by Alban.

Most previous research on the tracking loops of deeply-coupled integration was mainly based on relatively simple modeling and simulation. There was no literature available on the complete modeling for the INS branch in the INS-aided tracking loops, so there was no mature model supporting the design and evaluation of INS-aided tracking loops, such as the design of the tracking loop's bandwidth, selection of inertial sensors, implementation optimization of aiding information delay and quantitative analysis of tracking performance [6–15]. He *et al.* proposed a mathematical structure of the INS-aided delay lock loop (DLL) in 1998, shown in Figure 2a [9]: the INS branch is simply modeled as (1−*b*) / (*τs* +1), where *b* is the error of the IMU scalar factor and *τ* is delay time in the filter. However, the error of the IMU bias and the navigation error were not considered in the model. Alban proposed a mathematical structure of the INS-aided phase lock loop (PLL) in 2003, shown in Figure 2b [10]: the IMU is simply modeled as a low pass filter, which could not correctly reflect the error transformation of IMU; the INS information delay to tracking loops was not considered. Therefore, these models only demonstrated that satellite signal tracking performance could be enhanced with INS aiding, but could not be used for quantitative analysis to guide IMU selection, parameter optimization and quantitative analysis of INS-aided PLLs. In addition, since developing a deeply-coupled system requires the adjustment of the receiver internal structures, there were only limited cases of real-time hardware systems achieved by the co-operation of companies [14,15]. However, most research institutions use software platforms [16–18], resulting in literature being scant on the deeply-coupled system performance verification on embedded hardware platforms.

To provide a theoretical tool for IMU selection, parameter optimization and quantitative analysis of INS-aided PLLs, this paper builds the error transfer function of INS-aided PLLs based on a scalar-based architecture in Section 2, which reflect the relationships between all known error sources (including thermal noise, oscillator noise, INS errors and the delay of INS aiding information) and carrier phase tracking error. What needs to be emphasized is that INS errors are modeled in detail in this paper, which is proposed for the first time, compared to previous works. Based on that, it derives and analyzes the PLL steady-state error model before and after INS information assistance in Section 3, which can be used for the parameter design of INS-aided PLLs, IMU selection and hardware system development. Moreover, an integrated real-time deeply-coupled hardware prototype is developed in Section 4, in which the real-time running and the INS information delay are optimized. The proposed hardware prototype design is also unique compared to software systems in previous works. In Section 5, tests and analysis of PLL tracking performance in dynamic conditions are carried out based on a GPS/INS simulator and vehicle. Finally, the INS-aided PLLs on a deeply-coupled hardware prototype are summarized and concluded.

## 2. Error Transfer Function of INS-Aided PLLs

Compared with the DLL, the PLL is more sensitive to dynamic stress and much easier to lose locking, since the carrier wavelength is much shorter than the code chip length. Therefore, this paper studies the tracking performances of the PLL as an example. Error transfer function derivation is the precondition of the steady-state error modeling used for INS-aided PLLs design. To derive the error transfer function of INS-aided PLLs, its mathematical structure should be obtained based on the deeply-coupled system principle. In this section, the detailed architecture of the scalar-based deeply-coupled system will be introduced first. Then, the mathematical structure of INS-aided PLLs is proposed, reflecting the effects of all known error sources on tracking error. Based on that, the error transfer functions between all error sources and tracking error are modeled.

### 2.1. INS-Aided PLLs' Principle

**Figure 3.**
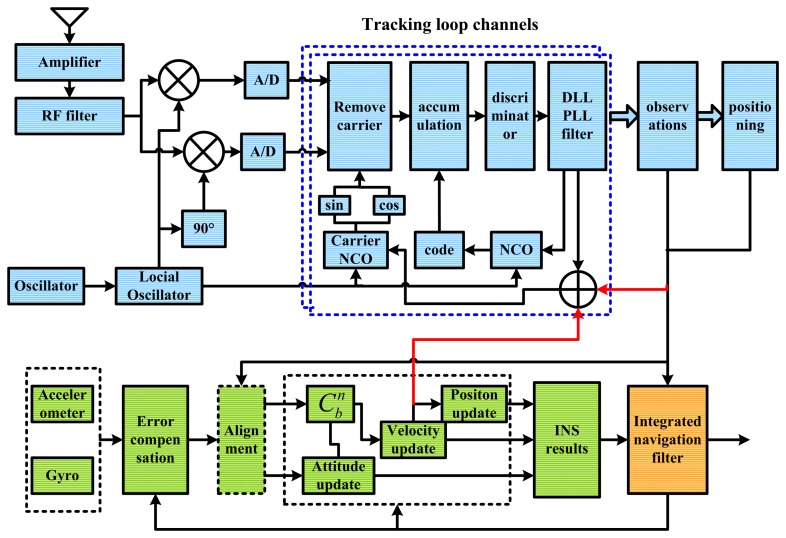
Detailed architecture of a scalar-based deeply-coupled system. DLL, delay lock loop.

With the GPS L1 single frequency receiver as example, Figure 3 shows the detailed components of the scalar-based deeply-coupled system. While the upper part represents the GPS receiver subsystem, the lower part is the inertial navigation subsystem. Compared with loosely-coupled integration and tightly-coupled integration, the INS information is sent to the receiver's baseband (red arrows in the figure), realizing the assistance to the receiver at the signal processing level. Compared with the vector-based deeply-coupled integration, the two subsystems are relatively independent in the scalar-based deeply-coupled integration, in which the internal structure is adjusted less. Since the receiver and INS are two kinds of navigation sensors, they can respectively measure the same vehicle motion information in different ways. As the INS and receiver's tracking loop are both used to compute the vehicle motion information, *i.e.*, the vehicle dynamics is commonly experienced by both the IMU and the tracking loop, the IMU measurement information could assist the tracking loop based on the concept of feed-forward [8].

**Figure 4.**
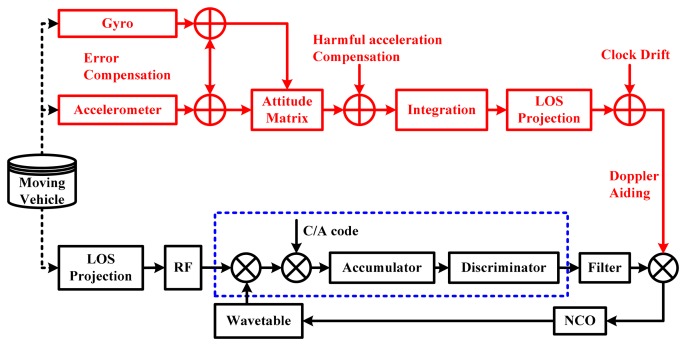
Principle of INS-aided PLLs. C/A code, coarse/acquisition-code.

Based on the scalar-based deeply-coupled system architecture, the principle of the INS-aided PLLs is shown in Figure 4. The PLL can track the carrier phase changing between a satellite and the vehicle, which measures the phase difference between local numerically controlled oscillator (NCO) and the input signal, by the discriminator converts the phase difference to the frequency by a low pass filter and adjusts the local NCO to follow the input signal. On the other hand, the vehicle movement information in the navigation frame can be directly measured by IMU. The measured velocity information is mapped to the line of sight (LOS) between the satellite and the vehicle to obtain the Doppler of relative motion, which combines with the receiver's clock drift, forming the Doppler aiding information. Therefore, with the aid of the INS feed-forward information, the tracking loop only needs to undertake the error of the INS aiding information, which significantly reduces the dynamic stress affecting the tracking loop.

Different error sources lead to different effects on the tracking loop. The error sources of the normal PLLs mainly include thermal noise, oscillator noise and dynamic stress. By contrast, the dynamic stress is replaced by the INS measurement error in the INS-aided PLLs. The INS measurement error could be divided into INS bias-type errors that are unrelated to dynamic and INS scale factor-type errors, which are related with the dynamics. In addition, the aiding information delay should be considered in the INS-aided PLLs. Hence, the error sources of the INS-aided PLLs include thermal noise, oscillator noise, INS bias-type errors, INS scale factor-type errors and aiding information delay. Moreover, the INS bias-type errors can be further divided into IMU sensor errors (constant bias, bias instability and white noise) and the initial navigation error (velocity error and attitude error) after correction of the GNSS/INS integration algorithm; the INS scale factor-type errors can be further divided into IMU scale factor error and IMU non-orthogonal error.

### 2.2. Error Transfer Function of INS-Aided PLLs

**Figure 5.**
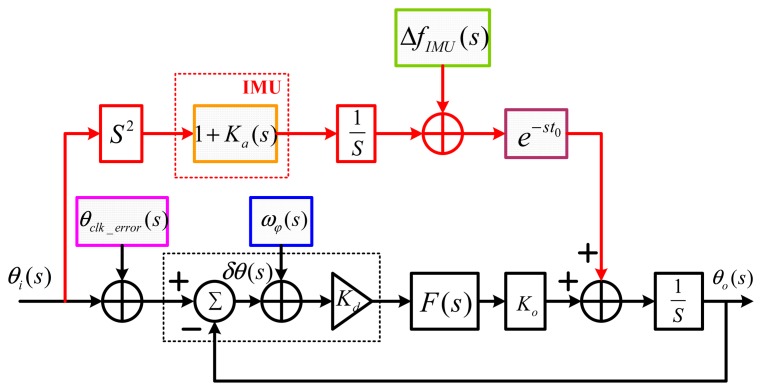
Mathematical structure of INS-aided PLLs.

According to the principle of INS-aided PLLs introduced in Section 2.1, the mathematical structure of the INS-aided PLLs is shown in Figure 5, in which the tracking error *δθ* is affected by all of the error sources simultaneously. As for a normal PLL, the effects of the thermal noise *ω_φ_* and oscillator noise *θ_clk_*___*_error_* on the tracking error are uncorrelated with each other. Besides, the error sources from the INS branch are physically independent from the PLL branch. Since the NCO is controlled by the sum of the PLL filter output and the INS aiding information, the relation of the tracking error caused by them is additive. According to the inertial navigation principle, the INS bias-type errors Δ*f_IMU_*, the INS scale factor-type errors *K_a_* and the INS aiding information delay *e*^−^*^st^*^0^ are independent of each other. Therefore, all of the error sources of the INS-aided PLLs are independent, and their effects on the tracking error are additive. All of the known major errors in the INS branch are considered in Figure 5, which is much more complete than the errors considered in Figure 2.

The normal PLL's error transfer function was derived from its mathematical structure [18]. The mathematical structure become more complex after INS aiding, and the INS-aided PLL's transfer function cannot be obtained based on Figure 5 directly. Since the effects of all of the error sources on the tracking error are additive, the error transfer function between each error source and the tracking error could be analyzed individually. Additionally, the error transfer function of the INS-aided PLLs could be obtained by the superposition of the effects of all of the error sources, which is written as Equation (1). This error transfer function is the precondition of the steady-state error modeling in the next section.

(1) $$\delta \theta \left(s\right)=\left(1-H\left(s\right)\right)\left(\theta_{\text{clk}\_\text{error}}\left(s\right)-K_{a}\left(s\right)\theta_{i}\left(s\right)-\frac{\Delta f_{\text{IMU}}\left(s\right)}{s}+\left(1-e^{-st_{0}}\right)\theta_{i}\left(s\right)\right)-H\left(s\right)\omega_{\varphi }\left(s\right)$$

where *H* (*s*) is the system transfer function of the normal PLL, which can be written as follows [19].

(2) $$H\left(s\right)=\frac{\frac{K_{d}K_{o}F\left(s\right)}{s}}{1+\frac{K_{d}K_{o}F\left(s\right)}{s}}$$

## 3. Steady-State Error Modeling and Analysis

The tracking threshold of the PLL is the maximum steady-state error for the receiver to keep PLL locked. Additionally, the receiver loses lock when the steady-state tracking errors exceed a certain boundary. Because the tracking loops are nonlinear, especially near the threshold regions, only Monte Carlo simulations under the combined dynamic and signal-to-noise radio (SNR) conditions can determine the true tracking performance. However, general rules that approximate the tracking errors can be used based on the steady-state error model of tracking loops. Although numerous tracking error sources are in both normal PLLs and INS-aided PLLs, it is sufficient as a rule of thumb to track thresholds to analyze only the dominant error sources. In this section, to compare the PLL performance before and after INS aiding, a normal second-order PLL steady-state error model is deduced based on previous research [1,20,21]. Then, INS-aided second-order PLL steady-state error model is proposed based on its error transfer function. The INS bias errors are considered in detail when the error model of the INS-aided PLL is built. Finally, the steady-state error characteristic of PLLs before and after INS aiding is compared based on their steady-state error models, which could guide the optimal bandwidth selection in the deeply-coupled system design.

### 3.1. Steady-State Error Modeling of Normal Second-Order PLLs

A conservative rule of thumb for tracking threshold is that the three-sigma jitter must not exceed one-fourth of the phase pull-in range of the PLL discriminator [1]. When the PLL two-quadrant arctangent discriminator is used, it has a phase pull in the range of 180°. Previous research shows that the dominant sources of phase error in a normal PLL are phase jitter and dynamic stress error; while the phase jitter is the root sum squared (RSS) of every source of uncorrelated phase error, such as thermal noise and oscillator noise. The dynamic stress error is a three-sigma effect and is additive to the phase jitter [1]. Therefore, the one-sigma rule threshold for the PLL for the two-quadrant arctangent discriminator is:

(3) $$\sigma_{\text{PLL}}=\sqrt{\sigma_{\text{tPLL}}^{2}+\sigma_{rv}^{2}+\sigma_{rA}^{2}}+\theta_{e}/3\leq 15\left({}^{\circ}\right)$$

where *σ_PLL_* is one-sigma tracking error of the normal PLL in degrees. *σ_tPLL_* is one-sigma thermal noise in degrees. *σ_rv_* is one-sigma vibration-induced oscillator jitter in degrees. *σ_rA_* is Allan variance-induced oscillator jitter in degrees. *θ_e_* is dynamic stress error in the PLL.

In the second-order PLL, the typical relationship of the noise bandwidth *B_n_* (Hz) and the natural radian frequency *ω_n_* (rad/s) is *B_n_* = 0.53*ω_n_* [1]. Therefore, the thermal noise jitter for the second-order PLL is computed as follows [1]:

(4) $$\sigma_{\text{tPLL}}=\frac{360}{2\pi }\sqrt{\frac{B_{n}}{C/N_{0}}\left[1+\frac{1}{2T_{coh}\cdot C/N_{0}}\right]}\left({}^{\circ}\right)$$

where *C* / *N*_0_ is the carrier-to-noise power expressed as a ratio (Hz) and *T_coh_* is coherent integration time (seconds).

The vibration-induced oscillator phase noise is a complex analysis problem. Assuming that all occurring vibrations are equally distributed across the entire frequency range, the vibration-induced oscillator phase jitter for the second-order PLL is written as follows [20]:

(5) $$\sigma_{rv}=180\sqrt{\frac{f_{0}^{2}K_{g}^{2}G_{g}}{2.67B_{n}}}\left({}^{\circ}\right)$$

where *K_g_* is the g-sensitivity of the oscillator, *G_g_* is the single-sided spectral density of vibration and *f*_0_ is carrier frequency.

The Allan deviation phase noise is caused by the drift of the receiver oscillator, which is determined by the oscillator's material and craft. The second-order PLL jitter due to Allan deviation phase noise is written as follows [20]:

(6) $$\sigma_{rA}=180\sqrt{2f_{0}^{2}\left[\frac{\pi^{2}h_{-2}}{\sqrt{2}{\left(1.89B_{n}\right)}^{3}}+\frac{\pi h_{-1}}{4{\left(1.89B_{n}\right)}^{2}}+\frac{h_{0}}{4\sqrt{2}\left(1.89B_{n}\right)}\right]}\left({}^{\circ}\right)$$

In the equation, the clock parameters *h*_−2_, *h*_−1_ and *h*_0_ represent the frequency stability of a certain oscillator.

Due to the permanent motion of the satellites and possible receiver motion, the PLL has to track the resulting signal dynamics. Signal dynamics is a major problem for non-static applications and principally degrades the PLL tracking performance, because it causes phase jitter. The second-order PLL tracking loop dynamic stress error can be expressed by [1].

(7) $$\theta_{e}=\frac{\Delta ℜ}{{\left(1.89B_{n}\right)}^{2}}\left({}^{\circ}\right)$$

where Δℜ is the maximum LOS acceleration dynamics (°/s^2^).

Therefore, the steady-state error model of the normal second-order PLL can be given based on Equations (3)–(7), and it is expressed by:

(8) $$\sigma_{\text{PLL}}=\frac{180}{\pi }\sqrt{\begin{array}{l} \frac{B_{n}}{C/N_{0}}\left(1+\frac{1}{2T_{\text{coh}}\cdot C/N_{0}}\right)+\frac{\pi^{2}f_{0}^{2}K_{g}^{2}G_{g}}{2.67B_{n}}\\ +2\pi^{2}f_{0}^{2}\left(\frac{\pi^{2}h_{-2}}{\sqrt{2}{\left(1.89B_{n}\right)}^{3}}+\frac{\pi h_{-1}}{4{\left(1.89B_{n}\right)}^{2}}+\frac{h_{0}}{4\sqrt{2}\left(1.89B_{n}\right)}\right) \end{array}}+\frac{\Delta ℜ}{3{\left(1.89B_{n}\right)}^{2}}\left({}^{\circ}\right)$$

As we know the running conditions of the receiver, including GNSS signal strength and the vehicle dynamics, the optimal bandwidth of the normal PLL can be calculated based on the error model in Equation (8), as described in [21]. Using the idea of the optimal bandwidth of the normal PLL, a tool for the bandwidth selection of the INS-aided PLLs can be proposed. Therefore, if the INS errors in the INS-aided PLLs are modeled in detail, an error model of the INS-aided PLLs, like Equation (8), could be built, which could truly reflect the effect of the INS error on the tracking loop physically and be used for the parameter optimization of the INS-aided PLLs.

### 3.2. Steady-State Error Modeling of INS-Aided Second-Order PLLs

Under vehicle dynamic conditions, Δ*f_IMU_*(*s*) are the main error sources in the INS branch. Hence, while Δ*f_IMU_*(*s*) is modeled in detail, which refers to our preliminary study in the literature [22], *K_a_*(*s*) and *e*^−^*^st^*^0^ are simply modeled as random constants in the INS-aided PLL error transfer function (Equation (1)). For the horizontal movement of vehicles, the lower the satellite elevation is, the larger the PLL mapping error from the INS is. Meanwhile, the north error and the east error of the INS are symmetrical in the north-east-down (NED) navigation frame. Here, we use the north direction as an example. If the vehicle is aligned with the NED navigation frame and moves toward the north, the PLL tracking a satellite at the north direction of the vehicle will map the largest error from the INS. The largest error of the aiding information should be considered for the parameter design of the PLLs. Therefore, without loss of generality, assume that the vehicle is aligned with the NED navigation frame and a satellite is at the north direction of the vehicle; Δ*f_IMU_*(*s*) can be expressed by:

(9) $$\begin{array}{cc} \Delta f_{\text{IMU}}\left(s\right) & =\frac{2\pi }{\lambda }\{\frac{1}{s}\cdot \left[\frac{b_{ax\_c}}{s}+\frac{GM_{ax}\left(0\right)+w_{GMax}\left(s\right)}{s+\frac{1}{T_{a}}}+w_{ax}\left(s\right)\right]\\ & +\frac{g}{s^{2}}\cdot [\frac{b_{gy\_c}}{s}+\frac{GM_{gy}\left(0\right)+w_{GMgy}\left(s\right)}{s+\frac{1}{T_{g}}}+w_{gy}\left(s\right)]+\frac{1}{s}\cdot \delta V_{N}(0)+\frac{g}{s^{2}}\cdot \phi_{E}(0)\} \end{array}$$

where *b_ax_*___*_c_* is the constant bias of the *X*-axis accelerometer (*i.e.*, forward direction), *b_gy_*___*_c_* is the constant bias of the *Y*-axis gyro (*i.e.*, pitch gyro), *GM_ax_*(0) + *w_GMax_*(*s*) is a first-order Gauss–Markov process representing the bias instability of the *X*-axis accelerometer, *GM_gy_*(0) + *w_GMgy_*(*s*) is a first-order Gauss–Markov process representing the bias instability of the *Y*-axis gyro, *w_ax_*(*s*) is the white noise of the *X*-axis accelerometer, *w_gy_*(*s*) is the white noise of the *Y*-axis gyro, *δV_N_*(0) is the north initial velocity error after correction of the GNSS/INS integration algorithm and *ϕ_E_*(0) is the east initial attitude error (*i.e.*, pitch error) after coupled navigation correction. Next, this detailed model of the INS bias errors Δ*f_IMU_*(*s*) is analyzed for the error modeling of the INS-aided PLL.

Since the error sources, including the INS bias-type errors and the INS scale factor-type errors, are corrected when the coupled navigation results are updated, the relationship between each error source and the tracking error in time domain should be analyzed in the update interval of coupled navigation. While the tracking errors caused by the error sources with a random constant feature are analyzed by transforming their transfer functions to the time domain, the tracking errors caused by the error sources with the noise feature are analyzed by Monte Carlo simulation.

Only considering the INS bias-type errors in Equation (1), taking Equation (9) into Equation (1), we get the concrete model reflecting the relationship between all of the INS bias-type errors and the phase tracking error. The influence of each INS bias-type error on the tracking error can be quantitatively analyzed from the concrete model. Analysis results in the literature [23] showed that, compared with the tracking error caused by other INS bias-type errors, the tracking error caused by the north initial velocity error after correction of the GNSS/INS integration algorithm *δV_N_*(0) was dominant. Therefore, using the tracking error caused by *δV_N_*(0) to express the steady-state error of INS-aided PLLs caused by the INS bias-type error sources:

(10) $$\theta_{\text{bias}}=360\frac{\delta V_{N}\left(0\right)}{\lambda \cdot e\cdot 1.89B_{n}}\left({}^{\circ}\right)$$

where *e* is the Euler number and *λ* is the carrier wavelength.

Since the INS scale factor-type errors are modeled as random constants, the steady-state error could be derived from its error transfer function. When the acceleration dynamics Δℜ appear, the INS scale factor-type errors bring frequency ramp estimation errors *K_a_* · Δℜ into the second-order PLL, which can generate a steady-state error. Therefore, the steady-state error of the INS-aided PLL caused by the INS scale factor-type errors can be expressed as:

(11) $$\theta_{K_{a}}=\frac{360}{2\pi }\frac{K_{a}\cdot \Delta ℜ}{{\left(1.89B_{n}\right)}^{2}}\left({}^{\circ}\right)$$

When the vehicle movement changes, the jitter brought into the second-order PLL caused by the aiding delay is the frequency step stimulus, which does not generate steady-state tracking error.

Since the phase error *θ_bias_* is uncorrelated with that due to thermal noise and oscillator noise, the steady-state error caused by *θ_bias_* is the geometric sum relationship to that caused by thermal noise and oscillator noise. Therefore, compared with Equation (3), the one-sigma rule threshold for the INS-aided PLL tracking loop is:

(12) $$\sigma_{\text{Aid}\_\text{PLL}}=\sqrt{\sigma_{\text{tPLL}}^{2}+\sigma_{rv}^{2}+\sigma_{rA}^{2}+\theta_{\text{bias}}^{2}}+\theta_{Ka}/3\leq 15\left({}^{\circ}\right)$$

The steady-state error model of the INS-aided second-order PLL can be given based on Equations (4)–(12), and it is expressed as:

(13) $$\sigma_{\text{Aid}\_\text{PLL}}=\frac{180}{\pi }\sqrt{\begin{array}{l} \frac{B_{n}}{C/N_{0}}\left(1+\frac{1}{2T_{\text{coh}}\cdot C/N_{0}}\right)+\frac{\pi^{2}f_{0}^{2}K_{g}^{2}G_{g}}{2.67B_{n}}\\ +2\pi^{2}f_{0}^{2}\left(\frac{\pi^{2}h_{-2}}{\sqrt{2}{\left(1.89B_{n}\right)}^{3}}+\frac{\pi h_{-1}}{4{\left(1.89B_{n}\right)}^{2}}+\frac{h_{0}}{4\sqrt{2}\left(1.89B_{n}\right)}\right)+\frac{4\pi^{2}\delta V_{N}^{2}\left(0\right)}{\lambda^{2}e^{2}{\left(1.89B_{n}\right)}^{2}} \end{array}}+\frac{60K_{a}\Delta ℜ}{\pi {\left(1.89B_{n}\right)}^{2}}$$

Just as the parameter optimization idea of the normal PLL, if the GNSS signal strength and the vehicle dynamics are known, the optimal bandwidth of the INS-aided PLLs could be calculated based on Equation (13). Comparing Equation (13) with Equation (8), after the INS aiding, the effect of the INS bias-type errors is added into the steady-state error model; but the steady-state error caused by the dynamic stress error is replaced by the INS scale factor-type errors, which is only a small portion of the original dynamic stress error. In the next part, the steady-state error characteristic of PLLs before and after INS aiding is compared in detail based on the steady-state error models. Meanwhile, the optimal parameters of the normal PLLs and the INS-aided PLLs could be obtained. Moreover, thanks to the detailed model of the INS branch, the INS initial velocity error after correction of GNSS/INS integration *δV_N_*(0) as the main factor of all of the INS bias-type errors is established in relation to the carrier phase tracking error shown in Equation (10), which has never been discussed in previous works.

### 3.3. Steady-State Error Analysis

In order to analyze the INS effect on the PLLs, a typical low-grade INS with the model of MTI-G and a typical medium-grade INS with the model of FSAS are selected as illustrations [24,25], with their initial velocity errors *δV_N_*(0) of about 0.02 m/s and 0.005 m/s, respectively, based on GNSS real-time kinematic (RTK)/INS integration testing. In addition, an oven controlled crystal oscillator (OCXO) is used with the parameters listed in Table 1.

**Figure 6.**
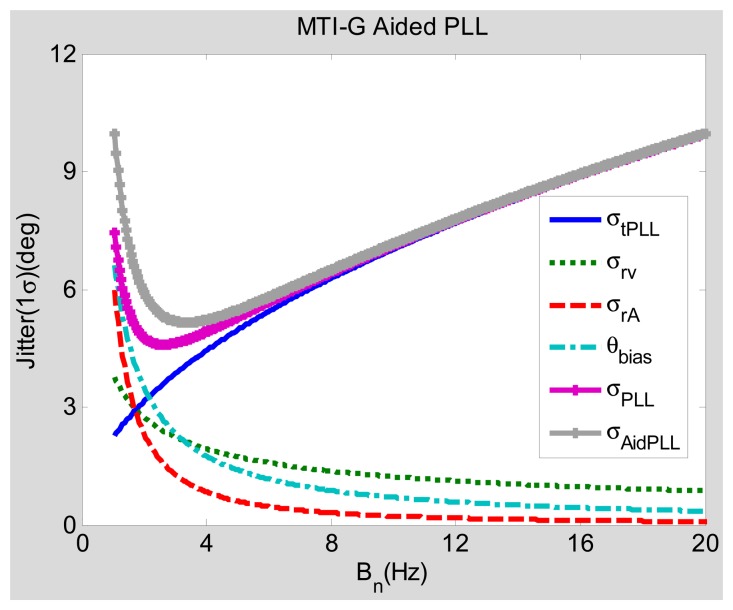
Tracking errors of MEMS INS-aided PLL under static conditions. MTI-G is a model of a typical low-grade INS.

**Figure 7.**
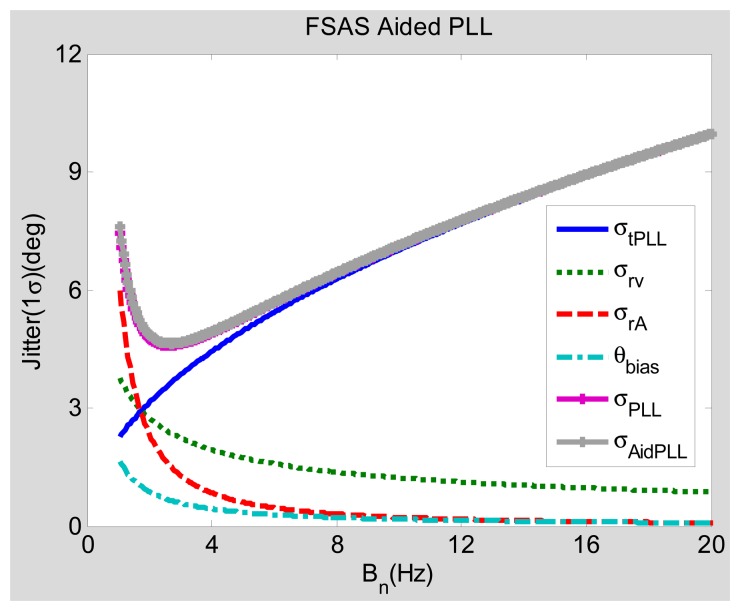
Tracking errors of medium-grade INS-aided PLL under static conditions. FSAS is a model of a typical medium-grade INS.

Since the INS bias-type errors are extra added error sources to the PLL, their impact to the steady-state error should be analyzed (under static conditions). Assuming that the acceleration Δℜ equals zero, Figures 6 and 7 show the steady-state tracking errors due to each individual error source and the total effect based on their steady-state error models (Equations (8) and (13)).

Figure 6 depicts the relationship between the bandwidth and the steady-state error of the low-grade INS (MTI-G)-aided PLLs with an integration time of 1 ms and 30-dB-Hz signal power under static conditions. When the horizontal axis represents the noise bandwidth *B_n_* (Bn = 0.53 *ω_n_*) of the PLL, the vertical axis is the steady-state phase tracking error in degrees. Similar to the trend of the steady-state error due to OCXO errors *σ_rv_, σ_rA_*, the steady-state error due to the INS bias-type errors *θ_bias_* rapidly decreases with the widening of the bandwidth. Hence, compared with the normal PLL's steady-state error *σ_PLL_*, the low-grade INS PLL's steady-state error *σ_Aid_*___*_PLL_* almost has no increase with a wide bandwidth. Only when the bandwidth is too narrow, *σ_Aid_*___*_PLL_* is just a little larger (less than one degree) than *σ_PLL_*. Therefore, when the acceleration equals zero (under static condition), the low-grade INS does not obviously increase the PLL's steady-state error, unless the bandwidth is excessively narrow.

Compared with Figure 6, Figure 7 shows the relationship of the bandwidth and the steady-state error of the medium-grade INS FSAS-aided PLL. The INS bias-type errors caused steady-state error *θ_bias_* of the PLL, with the medium-grade INS aiding being less than that with the low-grade INS aiding, especially when the bandwidth is narrow. Since the curve of *σ_AidPLL_* is in good agreement with that of *σ_PLL_*, the medium-grade INS does not obviously increase the static steady-state errors, even if the bandwidth is excessively narrow. Therefore, if the PLL is aided by a medium-grade INS, the effect of *θ_bias_* on the total phase tracking error can be ignored.

**Figure 8.**
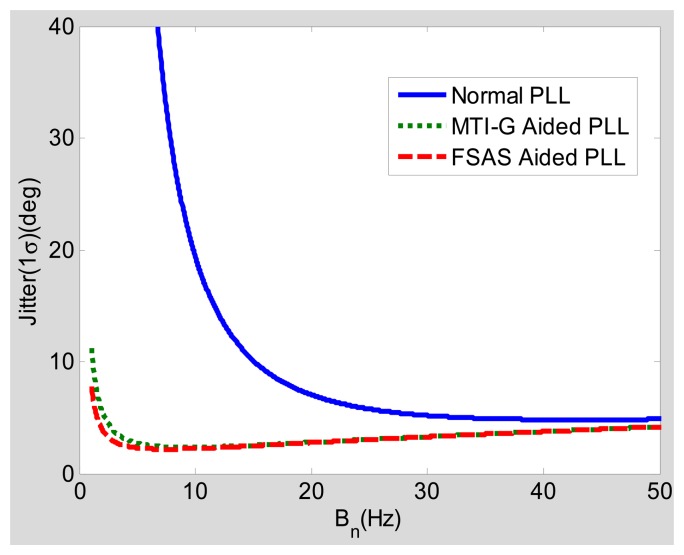
Steady-state tracking errors with the acceleration of 9.8 m/s^2^ and CNR of 40 dB-Hz.

**Figure 9.**
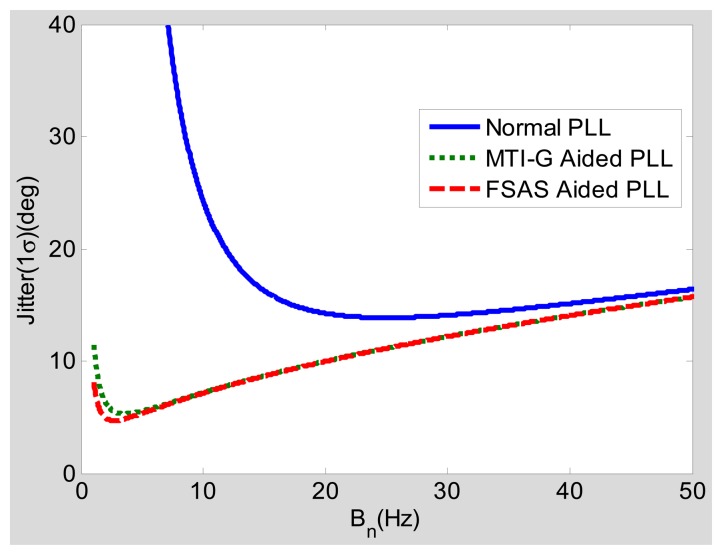
Steady-state tracking errors with the acceleration of 9.8 m/s^2^ and CNR of 30 dB-Hz.

Under dynamic conditions, the steady-state error caused by the dynamic stress should be considered. When the vehicle acceleration is 9.8 m/s^2^, the carrier to noise ratio (CNR) is 40 dB-Hz and the integration time is 1 ms, the relationships of the steady-state tracking errors and the bandwidths caused by normal second-order PLL, MTI-G-aided PLL and FSAS-aided PLL are as shown in Figure 8. It can be seen that the optimal bandwidth of the normal PLL is wider than 25 Hz, and its minimum tracking error is larger than five degrees; the optimal bandwidth of PLL with MTI-G or FSAS aiding is narrower than 5 Hz with the minimum tracking error being no more than three degrees. When the GNSS signal is attenuated to 30 dB-Hz, with other conditions unchanged, the result is as shown in Figure 9. Similar to the trend in Figure 8, the optimal bandwidth of the normal PLL must be wider than 20 Hz, due to the dynamic stress; and that of PLL with MTI-G or FSAS aiding can be narrower than 3 Hz. In addition, with the GNSS signal attenuated to 30 dB-Hz, the minimum tracking error of the normal PLL increases to 15 degrees; and that of the INS-aided PLL only increases to five degrees.

Steady-state error analysis shows that, with the assistance of INS information, the dynamic tracking bandwidth of the PLL could be narrower, and the dynamic tracking error can be reduced. It should be note that even the low-grade INS can significantly improve the PLL performance under dynamic conditions.

We summarize the steady-state error analysis as follows. The negative effect of the INS aiding, caused by the INS bias-type errors, is small and can be neglected, as shown in Figures 6 and 7. The positive effect of the INS aiding, by compensating for the dynamic stress of vehicle motions, is large and can improves the total steady-state phase tracking significantly, as shown in Figures 8 and 9. Hence, the steady-state error model could be used for optimizing the INS-aided PLL parameters, selecting inertial sensors and analyzing INS-aided PLL performance. Under static conditions with 30-dB-Hz signal power, the optimal bandwidth of the INS-aided PLLs is about 3 Hz, which is almost the same as that of the normal PLLs, and the static tracking error caused by INS bias-type errors is less than one degree. When the vehicle acceleration is 9.8 m/s^2^ and the signal power is 40 dB-Hz, the optimal bandwidth of the INS-aided PLLs is still about 3 Hz, which is much narrower than that of the normal PLLs (15 Hz). The dynamic tracking error of the INS-aided PLLs with optimal bandwidth is clearly lower than that of the normal PLLs, especially when the signal power drops to 30 dB-Hz. Therefore, the GNSS signal tracking sensitivity and accuracy of the PLLs can be proven after the INS aiding under dynamic conditions.

## 4. Design and Optimization of Hardware Prototype System

Based on the system modeling and error analysis in the previous sections, a scalar-based deeply-coupled system on an embedded platform is developed. The design and optimization methods of the hardware prototype are described in this section. The INS-aided PLLs in the deeply-coupled system will be designed based on the proposed steady-state error model on the embedded hardware platform.

### 4.1. Hardware Prototype Design

**Figure 10.**
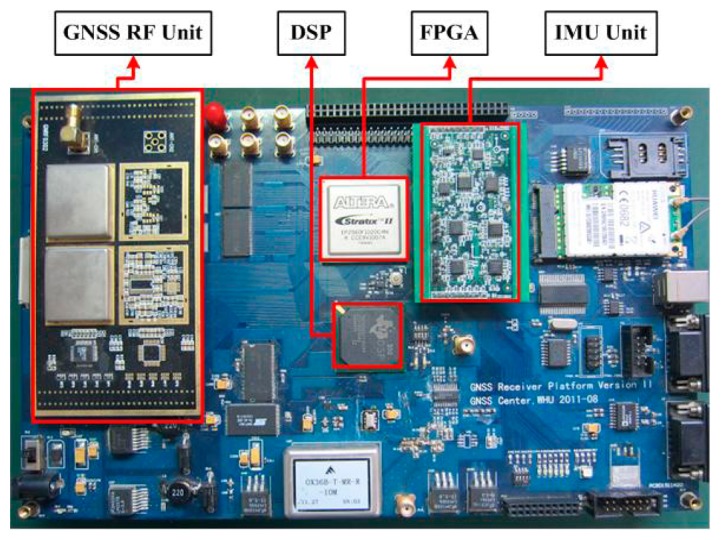
Hardware platform of a GNSS/INS deeply-coupled system. DSP means digital signal processor.

A hardware platform [26] with the processing core of a digital signal processor (DSP) plus a field programmable gates array (FPGA) is shown in Figure 10. While the DSP specializes in complex calculations and task scheduling, the coprocessor FPGA is good at high-speed digital signal processing in parallel and the interface control. The GNSS RF unit and the MEMS IMU unit are used to receive GNSS signals and inertial data, which are connected to the processor by the I/O of FPGA. The MEMS IMU on the prototype consists of a tri-axis accelerometer (LIS344ALH), a single-axis gyroscope (LPR510AL) and a double-axis gyroscope (LY510ALH). In addition, all units on the platform share the same clock, which can be TCXO, OCXO or other external clocks.

**Figure 11.**
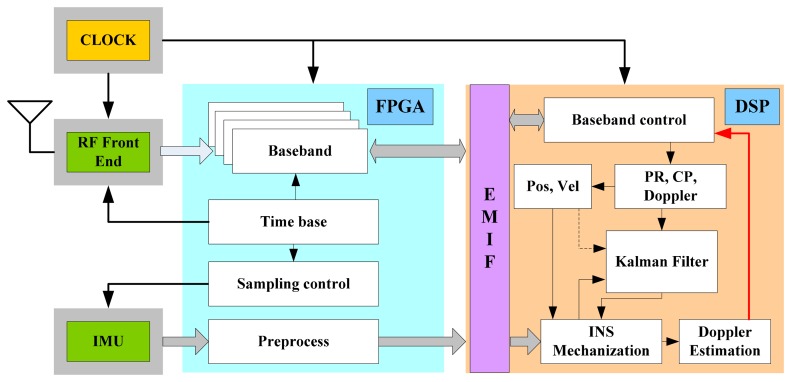
Integrated GNSS/INS deeply-coupled software architecture. PR represents pseudo-range, CP represents carrier-phase.

The scalar-based deeply-coupled system described in Figure 3 is implemented on the hardware platform, and its task assignment is shown in Figure 11. While the FPGA is responsible for GNSS IF data sampling, GNSS baseband correlators, IMU data sampling and preprocessing, *etc.*, DSP is used for GNSS baseband control, satellite positioning, INS mechanization, Kalman filter algorithm, the LOS Doppler estimation and tracking loop aiding. Using the sampling clock generated by the FPGA, the down-converted GNSS IF signal is digitized in the RF unit and sent to the baseband for digital signal processing. The correlators with high speed and multi-channels (including carrier NCO, code NCO, code generator, mixer and accumulator) are completed in the FPGA, and the timing module needs to be realized in the FPGA. The accumulating results are passed to DSP via the data bus in every interrupt, and the acquisition and tracking control of all channels are realized by the flexible DSP program. High-speed data exchange between the FPGA and DSP is carried out through the external memory interface (EMIF) of the DSP.

There are several advantages to implementing the deeply-coupled system on this hardware platform.

(i) All of the function units are triggered by the same clock, which is beneficial for time synchronization.
(ii) Different types of oscillators, IMUs and GNSS RF units can be chosen for a series of comparative experiments.
(iii) The high-speed interface is helpful for frequent data exchanging in the deeply-coupled system.
(iv) All of the software algorithms are realized on the same DSP, which is consistent with the integration feature of the deeply-coupled system.

### 4.2. Hardware Prototype Optimization

Different from the deeply-coupled system implemented in software receivers, the hardware prototype optimization copes with embedded software architecture, synchronization of IMU and GNSS data sampling and INS aiding information delay.

#### 4.2.1. Embedded Software Architecture

DSP needs to respond and process GNSS data, as well as IMU data simultaneously. Therefore, DSP should respond to two external interrupts, priority interrupt (Interrupt 1), used for receiving and processing GNSS correlator output, and priority interrupt (Interrupt 2) used for receiving and processing IMU raw data. Since the correlators update every millisecond, the interval time of Interrupt 1 must be less than 1 ms to receive each integration result. Considering the processor computing power and the maximal vehicle dynamics, the interval time of Interrupt 1 is set as 0.707 ms the Interrupt 2 as 50 ms.

When DSP enters an interrupt service routine, other interrupts are forbidden by default, whatever their priority. If the execution time of an interrupt exceeds 0.707 ms, some 0.707-ms interrupts will be missed. Therefore, interrupt priority control and interrupt nesting in the software are necessary. Interrupt 1 with a 0.2-ms execution time has the highest priority, and Interrupt 2 with a 10-ms execution time has the second highest priority. At the same time, interrupt nesting is allowed in the lower priority interrupt. Tests showed that the processor was able to handle all tasks completed within the tolerated time, even with abundant free time [26].

#### 4.2.2. Synchronization of Data Sampling

In the GNSS/INS applications, unknown timing errors between IMU and GNSS measurements have a significant influence on the data fusion performance of the Kalman filter. Additionally, the most effective method is to sample IMU data under the GNSS PPS (pulse per second) trigger, which could realize the time synchronization essentially. It needs two conditions: (i) the GNSS receiver can provide PPS; or (ii) the IMU sampling time could be controlled by an external signal. In this hardware prototype; the GNSS receiver subsystem could provide PPS, and the IMU module consists of gyroscopes and accelerometers with analogue interfaces, sampled by a multiplexing analog to digital converter (ADC) with controllable sampling time.

**Figure 12.**
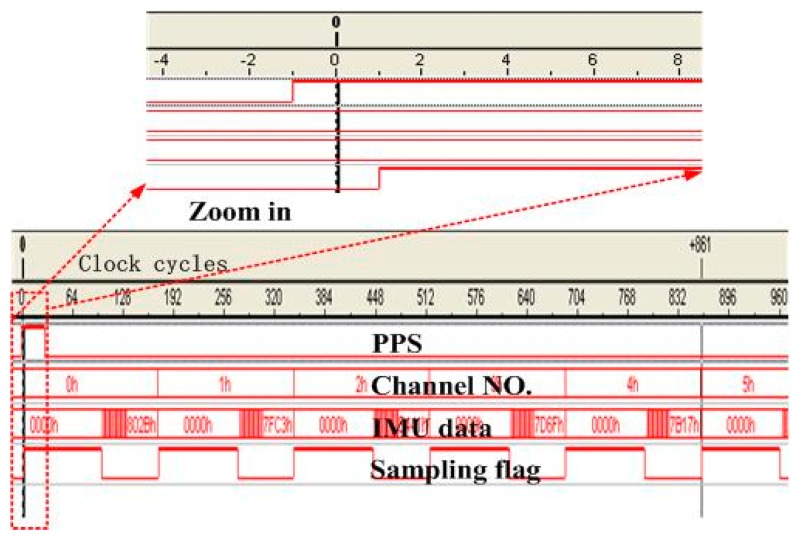
Sequence diagram of the IMU data sampling. PPS means pulse per second.

The IMU data sampling control module is designed in the FPGA, without additional cost of hardware [26]. Six signals from the sensors (three-axis gyroscopes and accelerometers) are sent to the input of the ADC, and the ADC is triggered by a 200-Hz pulse train to sample the IMU data. The PPS signal is generated in the time base module of the receiver to initialize a time-stamp counter. Then, the counter could generate the 200-Hz pulse train to trigger the ADC, and the time stamp is added to the IMU data. Figure 12 shows the sequence diagram of the IMU data sampling. The starting time of the first ADC channel conversion is delayed by two clock cycles compared to the PPS signal, with the first cycle using PPS rising edge detection and generating 200-Hz sampling pulses and the second cycle for triggering the conversion by the 200-Hz pulse train. Since the system clock is 39 MHz in the logic analyzer, the signal delay of Channel No. 0 is 0.05 μs. Other channels' signal synchronization errors are caused by their previous channel sampling time, and the longest delay is five-times the ADC sampling time. Since the shortest delay is 0.05 μs and the longest one is 22 μs, the effect of sampling time delay could be ignored for the deeply-coupled system, even in a highly dynamic environment.

#### 4.2.3. INS Aiding Information Delay

If the INS aiding information cannot be provided to the receiver tracking loop and reflect the vehicle dynamics in time, its contribution will be greatly degraded, especially with strong vehicle dynamics. To ensure the performance of the deeply-coupled system, the time delay of the aiding Doppler should be short enough.

**Figure 13.**
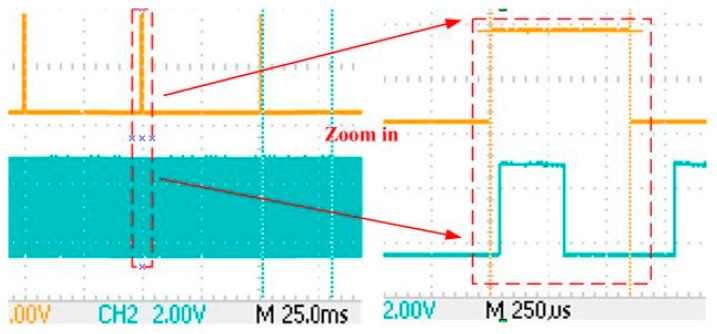
Time delay of the INS aiding data.

Data transmission is carried out between the FPGA and DSP in the integrated system. On the one hand, GNSS data and IMU data use the same transmission channel (EMIF), which causes basically the same delay. On the other hand, the EMIF can make communication efficient and seamless. Therefore, the processing time difference between loop filter output and INS aiding information is mainly caused by the different processing time of the discriminator, loop filter and INS mechanization, as well as Doppler estimation. In order to reduce the INS aiding information delay, the Doppler estimation is firstly executed once the INS mechanization is completed. Figure 13 shows the test result of adding data delay. While the operation time is about 382 μs, the transmission time is about 12 μs, which is negligible. Assume the dynamics of the platform is 100 m/s^2^; the Doppler change rate will be 525 Hz/s. Therefore, a delay of 0.4 ms causes a 0.21-Hz Doppler error, which has very little effect to the tracking loop.

Based on the design and optimization of the hardware prototype, the scalar-based deeply-coupled system was implemented on the platform in real time. With the guidance of the proposed steady-state error model, the INS-aided second-order PLL is designed on the embedded hardware platform. Next, the tracking performance evaluations of the second-order PLL before and after INS aiding will be carried out on the hardware prototype.

## 5. Tracking Performance Verification

To verify the effect of the INS aiding to carrier phase tracking performance for dynamic applications, comparison testing of normal PLLs and INS-aided PLLs is carried out on the hardware prototype under simulation and field vehicle scenarios.

The parameters of PLLs are selected based on the analysis results of the steady-state models given in Section 3. According to Figure 8, the optimal bandwidth of INS-aided PLLs is about 3 Hz when the vehicle acceleration is 9.8 m/s^2^, the bandwidth of INS-aided PLLs is set as 3 Hz. Figure 6 shows that the tracking error of normal PLLs under static conditions is also minimum when the bandwidth is set at about 3 Hz. However, the bandwidth of normal PLLs has to be widened to keep lock under motion conditions in practice, as shown in Figure 8. To verify the conclusion that the bandwidth can be set narrow enough under motion conditions only when the PLLs are aided by INS, the tracking performance of the normal PLLs with a bandwidth of 3 Hz is compared with the INS-aided PLLs with the same bandwidth. To demonstrate another conclusion that INS-aided PLLs could reduce the tracking error under motion conditions, the tracking error of the normal PLLs with a bandwidth of 10 Hz is compared with the INS-aided PLLs with a bandwidth of 3 Hz.

### 5.1. Simulator-Based Testing and Verification

**Figure 14.**
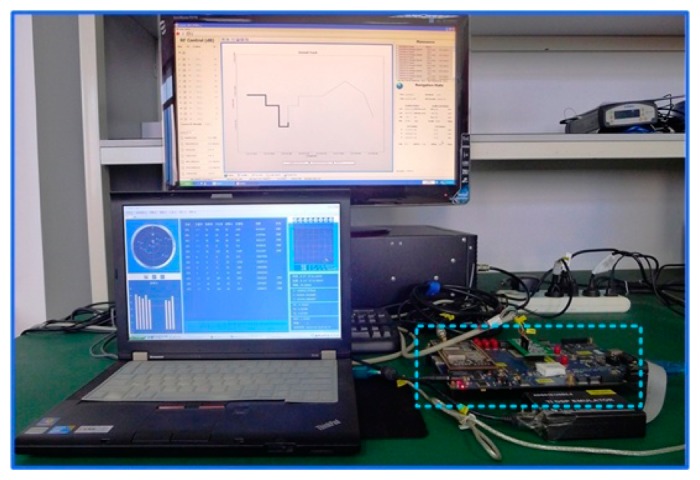
Setup of the simulation testing.

A GNSS/INS hardware signal simulator can generate typical scenarios with strict repeatability, well controlled motion states, less external disturbance and, most importantly, with perfect true values for error analysis. The simulation testing setup is shown in Figure 14. GPS L1 and IMU signals from the simulator are connected to the deeply-coupled hardware prototype through the RF module and the IMU module, respectively. Additionally, Table 2 shows the two typical IMU configuration parameters used in the simulation, one for low-grade MEMS IMU, the other for medium-grade IMU. To test the PLL's dynamic tracking performance, two sets of motion scenarios are designed, including the static, acceleration/deceleration, constant speed, turning, *etc.*, while the maximum acceleration is less than 5 m/s^2^ in the first set of motion scenarios, and the maximum acceleration is about 25 m/s^2^ in the second set of motion scenarios.

**Figure 15.**
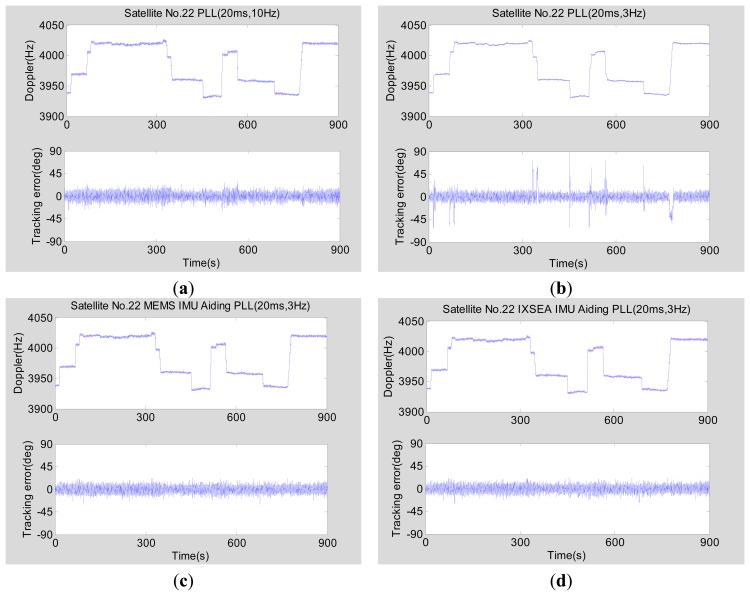
GNSS carrier phase tracking error in the lower dynamic scenario testing based on the simulator. (**a**) Normal PLL (20 ms, 10 Hz); (**b**) normal PLL (20 ms, 3 Hz); (**c**) MEMS INS-aided PLL (20 ms, 3 Hz); (**d**) medium-grade INS-aided PLL (20 ms, 3 Hz).

In the lower dynamic scenario (the maximum acceleration is less than 5 m/s^2^), the tracking error of pseudorandom noise (PRN) 22 as an example is analyzed with a CNR of 45 dB-Hz and an elevation of 20 degrees. Figure 15 shows the tracking errors of normal PLLs and INS-aided PLLs. While the upper part in each subfigure is the Doppler between the vehicle and satellite, which could reflect the vehicle movement toward the satellite, the lower part is the discriminator output, which could reflect the phase tracking error [27].

Figure 15a shows the tracking performance of the normal second-order PLL with an integration time of 20 ms and a bandwidth of 10 Hz. Since the vehicle acceleration is small and the bandwidth of the PLL is wide, the tracking error almost has no change when the vehicle movement changes. Figure 15b depicts the tracking error of the normal second-order PLL with an integration time of 20 ms and a bandwidth of 3 Hz. The overall magnitude of the tracking error does not significantly reduce compared with that in Figure 15a. That is because compressing the bandwidth cannot remove much thermal noise when the GNSS signal is strong, which can be explained by Equation (4). However, a larger error would appear once the vehicle movement changes due to the narrow bandwidth. The above phenomenon illustrates that a normal PLL is not suitable for a narrow bandwidth to suppress noise under dynamic conditions. As is seen in Figure 15c,d, with the MEMS INS or the medium-grade INS aiding, the tracking error of the PLL with an integration time of 20 ms and a bandwidth of 3 Hz does not increase when the vehicle accelerates/decelerates.

**Figure 16.**
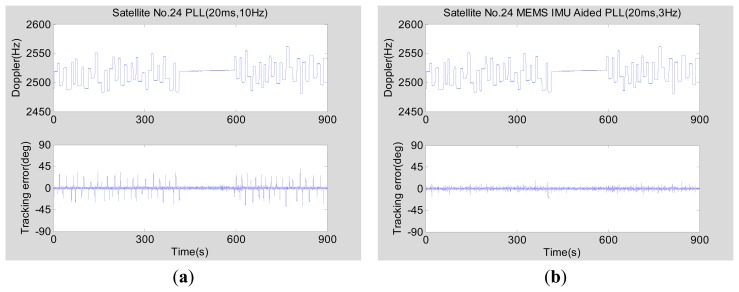
GNSS carrier phase tracking error in the higher dynamic scenario testing based on the simulator. (**a**) Normal PLL (20 ms, 10 Hz); (**b**) MEMS INS-aided PLL (20 ms, 3 Hz).

In the higher dynamic scenarios (the maximum acceleration is about 25 m/s^2^), the tracking error of PRN 24 as an example is analyzed with a CNR of 50 dB-Hz and an elevation of 34 degrees. Figure 16 shows the tracking errors of normal PLLs and INS-aided PLLs. Figure 16a shows the tracking performance of the normal second-order PLL with an integration time of 20 ms and a bandwidth of 10 Hz. Although the bandwidth is wide, the phase tracking error has a significant increase due to large dynamics. The above phenomenon illustrates that a normal PLL is not suitable for long time integration and a narrow bandwidth to suppress noise when the vehicle acceleration is large (more than 9.8 m/s^2^). As seen in Figure 16b, the phase tracking error of MEMS INS-aided second-order PLL with an integration time of 20 ms and a bandwidth of 3 Hz slightly increased because of the large dynamics.

The test results in Figures 15 and 16 are summarized in Table 3, which clearly shows the performance differences of various types of PLLs.

Comparing Line 2 and Line 3 in the table, the statistical results of the normal PLL and the INS-aided PLL with a bandwidth of 3 Hz under a CNR of 45 dB-Hz and an acceleration of 5 m/s^2^ show that the static tracking error of the normal PLL is slightly lower than that of the INS-aided PLL, which is because the INS bias-type errors are transferred to the PLL. However, the dynamic tracking error of the normal PLL is much larger than that of the INS-aided PLL, which is consistent with the model analysis result in Figure 8. Hence, the bandwidth of the PLL can be set narrow enough only when the PLL is aided by INS to respond to the vehicle dynamics, which verifies the model analysis conclusion in Section 3.3. Comparing the statistical results of the normal PLL with a bandwidth of 10 Hz (Line 1) with the INS-aided PLL with a bandwidth of 3 Hz (Line 3) under a CNR of 45 dB-Hz and an acceleration of 5 m/s^2^, the static and dynamic tracking errors of the INS-aided PLL are both lower than that of the normal PLL, which is consistent with the model analysis result shown in Figure 8. The advantage of the PLL with INS aiding would be more apparent with the GNSS signal attenuation, as shown in Figure 9. When the maximum acceleration is up to 25 m/s^2^, the dynamic tracking error of the normal PLL with a bandwidth of 10 Hz has significant increases. Therefore, the bandwidth of the normal PLL needs to be wider to reduce the dynamic error. On the contrary, the INS-aided PLL's dynamic tracking error only has a small increase, compared to its static tracking error. Therefore, the INS-aided PLL could reduce the carrier phase tracking error by compressing the bandwidth under motion conditions, which agrees with the analysis conclusion in Section 3.

Comparing Line 2 and Line 3, the static tracking error of the INS-aided PLL is slightly worse (not more than one degree) than that of the normal PLL, because of the INS bias-type errors, which is consistent with the analysis result shown in Figure 6. The dynamic tracking error of the PLL aided by the MEMS INS is almost the same as that aided by the medium-grade INS under vehicle dynamic conditions, which agrees with the model analysis results in Figures 8 and 9. All test results are consistent with the analysis results of the models, which verifies the feasibility of the proposed models in Section 3.

### 5.2. Vehicle-Based Testing and Verification

The objective of the field vehicle test is to validate the simulation results obtained in the previous section and to evaluate the real-time navigation performance of the deeply-coupled hardware prototype under realistic conditions.

**Figure 17.**
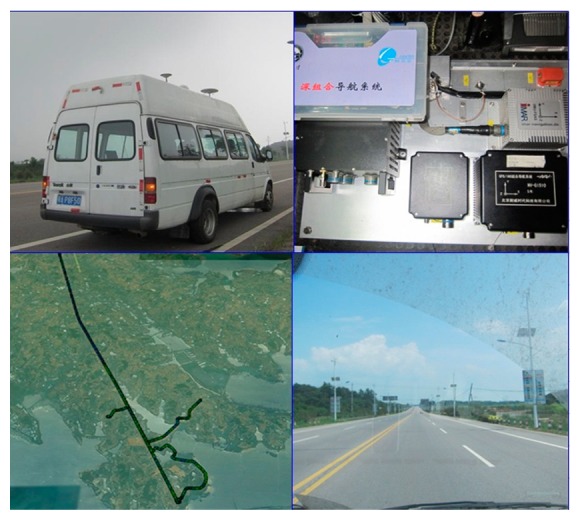
Setup of vehicle testing.

The vehicle dynamic testing was carried out in the suburbs of Wuhan under the open sky on 21 June 2013, to further verify the tracking performance. The test consists of a vehicle equipped with the self-built deeply-coupled prototype, medium-grade INS (FSAS), *etc*., and the open sky drive test, as shown in Figure 17. A GPS intermediate frequency (IF)/IMU raw data recording and playback unit is developed based on the hardware prototype, which cannot only replay GPS IF/IMU data to debug the deeply-coupled hardware prototype, but also can replace the onboard MEMS IMU data by other IMU data, such as FSAS, to achieve medium-grade INS-aided PLLs testing. The acceleration and deceleration of the road vehicle tests were less than 5 m/s^2^. Using PRN 13 as an example, the tracking error was analyzed with a CNR of 48 dB-Hz and an elevation of 45 degree.

**Figure 18.**
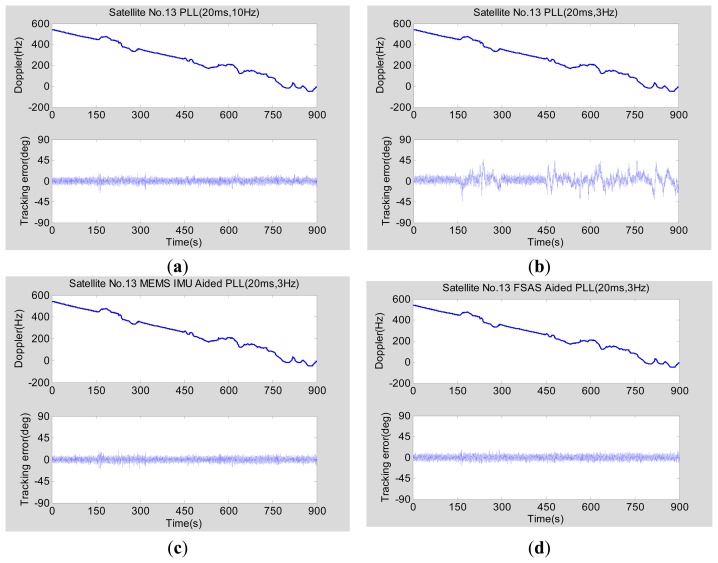
GNSS carrier phase tracking error testing based on the real vehicle test. (**a**) Normal PLL (20 ms, 10 Hz); (**b**) normal PLL (20 ms, 3 Hz); (**c**) MEMS INS-aided PLL (20 ms, 3 Hz); (**d**) medium-grade INS-aided PLL (20 ms, 3 Hz).

Figure 18 shows the tracking errors of normal PLLs and INS-aided PLLs. Figure 18a depicts the tracking error of a normal second-order PLL with an integration time of 20 ms and a bandwidth of 10 Hz. The tracking error almost has no change when the vehicle movement changes, similar to the simulator testing results in Figure 15a. Although the road vehicle dynamics are small (less than 5 m/s^2^), the dynamic tracking error increases significantly when the bandwidth of the normal PLL is compressed to 3 Hz, as shown in Figure 18b, similar to the simulator testing results in Figure 15b. Therefore, if the bandwidth of the normal PLL is compressed to enhance the tracking sensitivity of the carrier phase, its dynamic response error would increase, which is consistent with the normal PLL's error model, shown in Equation (8).

Figure 18c,d respectively depict the tracking error of the MEMS INS-aided PLL and the medium-grade INS-aided PLL with an integration time of 20 ms and a bandwidth of 3 Hz. The carrier phase tracking error does not increase with the vehicle dynamics, which is consistent with the theoretical analysis and the simulator test results. Furthermore, the assisting effect of MEMS IMU to the PLL is almost as good as the medium-grade IMU aiding effect. Therefore, the vehicle test results further validate the performance of INS-aided PLL designed based on the proposed steady-state error model on the deeply-coupled hardware prototype.

INS-aided PLLs can reduce the tracking error, and the navigation precision should be improved accordingly. This is verified by comparing the velocity errors of integrated navigation using the Doppler measurement from the PLLs without and with MEMS INS aiding. Table 4 shows the performance differences of the integrated navigation results without and with INS aiding. The velocity errors are clearly smaller when the PLL is aided by INS, as expected. Therefore, the INS-aided loops in deeply-coupled systems can improve the navigation performance.

## 6. Conclusions

The GNSS/INS deeply-coupled system can realize INS-aided GNSS PLLs and improve the dynamic tracking performance of the GNSS receiver. Based on the principle of the scalar-based deeply-coupled system, this paper proposes comprehensive error models and analyzes the detailed steady-state error model of the INS-aided PLLs, which can be used to guide IMU selection, parameter optimization and quantitative error analysis of INS-aided PLLs, compared to previous works of rough models for qualitative analysis. Then, the real-time deeply-coupled prototype based on a hardware platform was developed and optimized, which is unique and convincing compared to previous software receiver implementations. Finally, the tracking performances of normal PLLs and INS-aided PLLs are tested and compared through simulation and vehicle tests. The test results show that the dynamic tracking error of INS-aided PLL is much lower than that of the normal PLLs, compared with that of the normal PLLs, but its static tracking error is no more than one degree bigger, which is consistent with the proposed steady-state error model in this paper. The test results also verified that low-cost MEMS IMU performs as well as medium-grade INS in aiding PLLs. Moreover, the final navigation performance can be improved in terms of the INS-aided GNSS tracking loops. The proposed error model and the developed deeply-coupled hardware prototype in this paper can be further applied to the high-sensitivity and anti-interference GNSS receiver design and optimization under dynamic conditions.

## Figures and Tables

**Table 1.** OCXO parameters.

*K_g_*	*G_g_*	*h*_−2_	*h*_−1_	*h*_0_
1e−10 (1/g)	0.05 (g^2^/Hz)	2.51e−22	2.51e−23	2.51e−26

**Table 2.** IMU configuration parameters in the simulator. IXSEA is the model of a typical medium-grade INS.

**Parameters**	**Low-Grade IMU (MEMS)**	**Medium-Grade IMU (IXSEA)**
Gyro bias (deg/h)	36	0.05
Gyro white noise (deg/√h)	3.0	0.003
Gyro scale factor (ppm)	300	30
Accelerometer bias (mGal)	2000	100
Accelerometer white noise (m/s/√h)	0.12	0.09
Accelerometer scale factor (ppm)	300	40

**Table 3.** Standard deviations of carrier phase tracking errors (in degrees) for PLLs.

**1**	**PLL Type**	**Testing Conditions**	**Static Portions**	**Motion Portions**	**All Portions**
**2**	Normal PLL 20 ms, 10 Hz	Acc = 5 m/s^2^ CNR = 45 dB-Hz	6.0	7.9	6.3
**3**	Normal PLL 20 ms, 3 Hz	Acc = 5 m/s^2^ CNR = 45 dB-Hz	5.1	21.0	8.7
**4**	MEMS-aided PLL 20 ms, 3 Hz	Acc = 5 m/s^2^ CNR = 45 dB-Hz	5.4	6.1	5.5
**5**	IXSEA-aided PLL 20 ms, 3 Hz	Acc = 25 m/s^2^ CNR = 45 dB-Hz	5.2	6.0	5.3
**6**	Normal PLL 20 ms, 10 Hz	Acc = 25 m/s^2^ CNR = 50 dB-Hz	1.4	18.2	6.6
**7**	Normal PLL 20 ms, 3 Hz	Acc = 25 m/s^2^ CNR = 50 dB-Hz		Lose lock	
**8**	MEMS-aided PLL 20 ms, 3 Hz	Acc = 25 m/s^2^ CNR = 50 dB-Hz	1.3	2.1	1.7

**Table 4.** Standard deviations of velocity errors (in m/s) of integrated navigation.

**PLL Type**	**Vel_N**	**Vel_E**	**Vel_D**
Normal PLL (20 ms, 10 Hz)	0.040	0.040	0.127
MEMS aided PLL (20 ms, 3 Hz)	0.018	0.018	0.046
IXSEA aided PLL (20 ms, 3 Hz)	0.011	0.011	0.025
